# Intratumor heterogeneity: models of malignancy emergence and evolution

**DOI:** 10.18699/VJGB-23-94

**Published:** 2023-12

**Authors:** R.A. Ivanov, S.A. Lashin

**Affiliations:** Institute of Cytology and Genetics of the Siberian Branch of the Russian Academy of Sciences, Novosibirsk, Russia; Institute of Cytology and Genetics of the Siberian Branch of the Russian Academy of Sciences, Novosibirsk, Russia Novosibirsk State University, Novosibirsk, Russia

**Keywords:** cancer, evolution, heterogeneity, злокачественные опухоли, эволюция, гетерогенность

## Abstract

Cancer is a complex and heterogeneous disease characterized by the accumulation of genetic alterations that drive uncontrolled cell growth and proliferation. Evolutionary dynamics plays a crucial role in the emergence and development of tumors, shaping the heterogeneity and adaptability of cancer cells. From the perspective of evolutionary theory, tumors are complex ecosystems that evolve through a process of microevolution influenced by genetic mutations, epigenetic changes, tumor microenvironment factors, and therapy-induced changes. This dynamic nature of tumors poses significant challenges for effective cancer treatment, and understanding it is essential for developing effective and personalized therapies. By uncovering the mechanisms that determine tumor heterogeneity, researchers can identify key genetic and epigenetic changes that contribute to tumor progression and resistance to treatment. This knowledge enables the development of innovative strategies for targeting specific tumor clones, minimizing the risk of recurrence and improving patient outcomes. To investigate the evolutionary dynamics of cancer, researchers employ a wide range of experimental and computational approaches. Traditional experimental methods involve genomic profiling techniques such as next-generation sequencing and fluorescence in situ hybridization. These techniques enable the identification of somatic mutations, copy number alterations, and structural rearrangements within cancer genomes. Furthermore, single-cell sequencing methods have emerged as powerful tools for dissecting intratumoral heterogeneity and tracing clonal evolution. In parallel, computational models and algorithms have been developed to simulate and analyze cancer evolution. These models integrate data from multiple sources to predict tumor growth patterns, identify driver mutations, and infer evolutionary trajectories. In this paper, we set out to describe the current approaches to address this evolutionary complexity and theories of its occurrence.

## Abstract (ru)

Рак – сложное и гетерогенное заболевание, характеризующееся накоплением генетических изменений, которые приводят к неконтролируемому росту и пролиферации клеток. Эволюционная динамика играет решающую роль в возникновении и развитии раковых опухолей, формируя гетерогенность и адаптивность раковых клеток. С точки зрения теории эволюции опухоли представляют собой сложные экосистемы, которые развиваются в процессе микроэволюции под воздействием генетических мутаций, эпигенетических изменений и факторов микроокружения опухолей. Такая динамичная природа опухолей создает значительные проблемы для эффективного лечения рака, и ее понимание необходимо для разработки эффективных и персонализированных методов лечения. Раскрывая механизмы, определяющие гетерогенность опухоли, исследователи могут выявить ключевые генетические и эпигенетические изменения, которые способствуют прогрессированию опухоли и устойчивости к лечению. Эти знания позволяют разрабатывать инновационные стратегии воздействия на конкретные клоны опухоли, минимизируя риск рецидива и улучшая результаты лечения пациентов. Для изучения эволюционной динамики рака ученые используют широкий спектр экспериментальных и вычислительных подходов. Традиционные экспериментальные методы включают в себя геномное профилирование, такое как секвенирование нового поколения и флуоресцентная гибридизация in situ, и позволяют выявлять соматические мутации, изменения числа копий генов и структурные перестройки в геномах раковых опухолей. Помимо того, методы одноклеточного секвенирования стали мощным инструментом для изучения внутриопухолевой гетерогенности и отслеживания клональной эволюции. На основании экспериментальных данных разрабатываются вычислительные модели и алгоритмы для моделирования и анализа эволюции рака. Эти модели объединяют данные из различных источников для предсказания закономерностей роста опухоли, выявления драйверных мутаций и построения эволюционных деревьев развития раковых клеток. В настоящей работе мы поставили задачу описать существующие на сегодняшний день подходы к изучению эволюционной динамики развития рака и теории ее возникновения

## Evolutionary models of cancer

Cancer is a complex disease caused by the accumulation of genetic and epigenetic changes in normal cells, resulting in uncontrolled cell growth and tumor formation. Over the past few decades, it has become increasingly apparent that tumors are not static entities, but rather dynamic systems that undergo continuous evolution (Nowell, 1976; Merlo et al., 2006; Besse et al., 2018; Hausser, Alon, 2020; Vendramin et al., 2021). This evolutionary process shapes the heterogeneity and adaptability of cancer cells, posing significant challenges to effective cancer treatment. Tumor heterogeneity refers to the presence of different cell types in a tumor, commonly described as clones. In the context of oncology and evolutionary biomedicine, a clonal population is defined as a group of cancer cells that share a common origin and have similar genetic alterations. As these cells divide and accumulate additional mutations, they form separate clonal subpopulations in the tumor. This heterogeneity can manifest itself in various ways, such as differences in cell morphology (Meacham, Morrison, 2013; Robertson-Tessi et al., 2015; Haffner et al., 2021), differential gene expression of individual clones (Lüönd et al., 2021; Zhao et al., 2022), or their functional characteristics.

Clonal populations in cancer are commonly viewed as analogous to different species in the context of evolutionary biology (Vendramin et al., 2021). In the same way that different species evolve and adapt to their environment over time, clonal populations in a tumor evolve and adapt to their microenvironment. Genetic alterations emerging in these populations confer advantages or disadvantages in terms of growth, survival, and response to therapy, leading to selection and dominance of certain clones in the tumor.

Tumor heterogeneity represents a major treatment challenge because it can contribute to resistance to therapy, tumor recurrence after surgery, and the progression of metastasis (Morris et al., 2016). Currently, there are several theories regarding the mechanisms of the heterogeneity emergence in tumors.

The theory of clonal evolution is one of the earliest and most widely accepted theories that explains the occurrence of cancer heterogeneity. According to this theory, tumors originate from one or more transformed cells, the descendants of which acquire additional genetic mutations over time. These mutations lead to the formation of distinct clones with unique phenotypic characteristics. As the tumor grows, clones with advantageous traits are selected, resulting in the expansion and prevalence of these clones in the tumor population or their co-existence in the tumor depending on the type of cancer

The concept of clonal evolution includes several models – linear, branching, and punctuated. In the linear model, mutations are acquired in a linear progression leading to more malignant stages of cancer (Fearon, Vogelstein, 1990). In the linear evolution model, new driver mutations provide such a strong selective advantage that they outcompete all previous clones due to the selective sweeping that occurs during tumor evolution. In the branching evolution model, clones diverge from a common ancestor and develop in parallel in a tumor tissue, giving rise to multiple clonal lineages (Gawad et al., 2014; Vosberg, Greif, 2019). In contrast to linear evolution, in the branching model of evolution, selective sweeps are rare, and multiple clonal populations evolve simultaneously because they all have increased adaptability. In this model, the magnitude of intratumor heterogeneity will fluctuate during tumor progression, but multiple clones are expected to be present at any given time of tumor sampling.

The neutral evolution model challenges the traditional view that all genetic alterations in cancer confer a selective advantage. According to this theory, most genetic mutations in cancer are neutral or nearly neutral, that is, they have no significant effect on tumor fitness (Williams et al., 2016; Furukawa, Kikuchi, 2020). Instead, the occurrence of heterogeneity is caused by random genetic drift, where neutral mutations randomly accumulate in different clones. Over time, these neutral mutations can become fixed within clones, leading to the observed intratumor heterogeneity.

It is worth noting that this theory is compatible with another popular theory of mutation accumulation – punctuated evolution, mentioned earlier in the text. According to this hypothesis, cancer cells are Goldschmidt’s “hopeful monsters” (Graham, Sottoriva, 2017) – in which gradual and non-displayed changes in the genome lead to dramatic changes in the phenotype. Such a principle is evident in neoplasms in particular, since there are no obvious intermediate stages between healthy tissue and primary tumors. The intervals between the jumps, however, most likely represent the stages of neutral evolution. According to the same theory of punctuated evolution, the populations themselves may be in some kind of equilibrium with each other, maintaining several populations of clonal cancer cell lines in the tumor. After some time, one of the populations becomes a “hopeful monster” and in the case of a fitness-enhancing mutation, these clones occupy a larger part of the tumor, displacing the less adapted ones and increasing the size of the tumor itself.

Importantly, a number of studies have been reported that show that the development of an individual tumor does not necessarily follow a single pattern of clonal evolution and it can change during its development. Presumably, in the early stages of tumor development, it develops according to the linear evolution model, and once the tumor starts to actively grow, it switches to the branching model (Durrett et al., 2011; Vosberg, Greif, 2019). Moreover, several papers have shown that tumor evolution can follow both branching and punctuated models simultaneously – when clones with gene copy number changes follow the punctuated model and clones with point mutations follow the branching model (Baca et al., 2013; Wang et al., 2014).

Another common theory on the origin of heterogeneity is the cancer stem cell theory, which suggests that tumors are hierarchically organized structures and only a small population of cancer stem cells (CSCs) determines tumor growth and heterogeneity (Reya et al., 2001; Lee et al., 2022). CSCs have the ability to self-renew and differentiate, similar to normal stem cells. These cells are capable of generating both other CSCs and non-CSC progeny, which in theory contributes to the cellular diversity seen in tumors. An important aspect of this theory is the hierarchy of cancer cells – normal cancer cells are incapable of differentiation and somatic mutations in them have a less significant clinical effect due to a lower ability to reproduce, while the main pathological significance is due to CSCs with different degrees of pluripotency. The occurrence of heterogeneity in this model is explained by asymmetric division of CSCs, which can lead to the appearance of different CSC clones with different phenotypic properties. It is worth noting that so far CSCs have only been found in a limited number of tumor types, particularly in hematologic tumors (Bonnet, Dick, 1997; Zarzynska, 2017; Hata et al., 2018; Lee et al., 2022), but in these instances they may be a major factor in malignant tumor recurrence after treatment (Walcher et al., 2020).

The theory of microenvironmental selection suggests that the tumor microenvironment plays an important role in shaping tumor heterogeneity. The interaction between cancer cells and the surrounding microenvironment, which includes immune cells, stromal cells, and extracellular matrix components, may exert selective pressure on tumor cells (Augustin et al., 2020). Microenvironmental factors such as hypoxia, inflammation, and nutrient availability can influence tumor growth, angiogenesis, and metastasis (Mumenthaler et al., 2015; Roma-Rodrigues et al., 2019). This selective pressure favors the survival and reproduction of specific clones with advantageous traits that allow them to adapt to the microenvironment.

Among the factors of the microenvironment, the immune system plays a particularly important role. The action of immune cells has a double function in cancer development: it can both inhibit tumor growth and promote tumor progression. Immune checkpoint mechanisms recognize and destroy cancer cells, preventing tumor formation. However, tumors can evade the immune response through a variety of mechanisms, leading to the immune response acting as a natural selection factor for clonal populations and thus selecting the most resistant clonal populations with altered antigens, which directly affects the severity of the disease and the efficacy of immunotherapy.

Finally, the theory of epigenetic plasticity suggests that, in addition to genetic abnormalities, epigenetic alterations also play a significant role in causing tumor heterogeneity (Flavahan et al., 2017; Yao et al., 2020). Epigenetic modifications, such as DNA methylation and histone modifications, can dynamically regulate gene expression patterns and cellular phenotypes. According to this theory, cancer cells possess an epigenetic landscape plasticity that allows for reversible and dynamic changes in gene expression. These epigenetic changes can give rise to different clones with distinct phenotypic characteristics, contributing to intratumor heterogeneity.

## Approaches to the study of evolutionary characteristics in heterogeneous tumors

To study the evolutionary features of heterogeneous tumors, it is imperative for the researcher to be able to qualitatively and quantitatively assess different clonal populations. In the next section, we present a number of analysis methods that are currently used to study tumor heterogeneity.

The population genetics approach is one way to theoretically study heterogeneous tumor communities. According to population genetics, the evolution of a population relies on two factors: the mutation rate and the effective population size. The mutation rate refers to the expected number of genetic mutations per individual replication event and directly impacts the diversity within a population. The effective population size determines the population’s capacity to maintain this diversity. In tumors, the effective size is defined as the total number of cancer cells, but it is also possible to exclude some groups of cancer cells from this number – if, for example, a CSCinduced tumor is modeled, which would be the main cause of tumor growth. Of course, such an approach requires the use of single-cell sequencing of tumors. Due to the complexity and high cost of this method, classical population genetics analysis has only been performed in a few papers so far (Navin, 2015; Losic et al., 2020; Heinrich et al., 2021; Deng et al., 2023).

Since single-cell sequencing methods have only recently become available, much of the work has focused on studying heterogeneity using bulk next-generation sequencing methods on tumor samples. This approach has an obvious problem: it is difficult to directly identify the clonal architecture of a tumor in the data obtained from such samples. Therefore, using this approach, researchers have to make certain assumptions and modifications to experimental methods. One of them is to increase the sequencing depth to estimate the frequencies of mutant alleles (Koh et al., 2021). To analyze tumor populations, statistical methods are used to normalize these frequencies and cluster genotypes to identify identical clonal populations. Diversity characterizations like the Shannon diversity index and Simpson index are often employed in such studies. However, a drawback of this approach is its inability to distinguish between populations if they have similar mutant allele frequencies.

Another modification is multiregional sequencing, in which samples are collected from multiple tumor sites. In particular, this method allows us to assess the difference in heterogeneity in patients with multiple metastatic tumors, which in the context of diversity can be perceived as a population of clones with prolonged physical isolation.

The most promising techniques for experimental assessment of heterogeneity are methods of single cell analysis, as they allow us to judge the individual differences of clones at the genetic and phenotypic levels. Immunofluorescence in situ hybridization (iFISH) is one such technique. Through the use of fluorescently labeled DNA probes that hybridize with complementary target sequences, FISH allows the detection of genetic alterations, chromosomal rearrangements and gene amplifications with high specificity and sensitivity. In situ FISH (iFISH) is the implementation of FISH directly on tissue sections while preserving the spatial organization of cells in the tumor microenvironment (Gertz et al., 2016). However the iFISH method is low-throughput and does not allow for the investigation of heterogeneity at the full-genome level.

In contrast to the method described above, single-cell sequencing (scDNA-seq and scRNA-seq) allows us to determine the pattern of genetic diversity, gene expression in each individual cell and decipher its intercellular signaling networks. These methods provide a clear picture not only of the mechanisms of intratumor heterogeneity, but also of intercellular interactions through ligand-receptor signaling.

## Conclusion

Understanding the evolution and heterogeneity of malignant tumors is crucial for improving cancer diagnosis and developing treatment strategies. Many molecular genetic techniques, with their advantages and disadvantages, have been developed to study the genetic and phenotypic characteristics of cancer clone populations. Next-generation sequencing can provide a comprehensive view of the genomic landscape of a tumor, but there is a risk of missing rare clones. Single-cell sequencing can identify rare clones and reconstruct clonal lineages, but is technically challenging and expensive. Methods such as iFISH provide spatial information but have limited target coverage and are low throughput.

Based on the data obtained using such methods, various models have been proposed to explain the dynamic nature of tumor evolution, including models of clonal evolution, cancer stem cells, models of microenvironmental impact, and epigenetic factors. Each of them provides valuable insights into the mechanisms behind tumor heterogeneity and the emergence of drug resistance

Moreover, the development of mathematical and computational models of clonal evolution and algorithms for analyzing large-scale genomic data could enhance the ability to interpret and extract meaningful information from complex datasets of malignancies. These tools would potentially allow researchers to identify key driver events, track evolutionary dynamics, and more accurately predict the effects of treatment.

## Conflict of interest

The authors declare no conflict of interest.
